# Biochemical osteomalacia in adults undergoing vitamin D testing in the North-East of Scotland

**DOI:** 10.1177/00045632251315671

**Published:** 2025-01-25

**Authors:** Angus D Macleod, Mark J Bolland, Andrew Balfour, Andrew Grey, Josh Newmark, Alison Avenell

**Affiliations:** 1 Institute of Applied Health Sciences, 1019 University of Aberdeen, Aberdeen, Scotland; 2 Department of Medicine, 1415 University of Auckland, Auckland, New Zealand; 3 Department of Clinical Biochemistry, Aberdeen Royal Infirmary, 1015 NHS Grampian, Aberdeen, Scotland; 4 Health Services Research Unit, Institute of Applied Health Sciences, 1019 University of Aberdeen, Aberdeen, Scotland

**Keywords:** Vitamin D, osteomalacia, hypocalcaemia, deficiency, insufficiency

## Abstract

**Background:**

International guidelines give greatly varying definitions of 25-hydroxyvitamin D (25OHD) insufficiency and deficiency. Vitamin D testing is increasing despite 2016 UK guidance for adults advising routine vitamin D supplementation October-March and year-round for high risk groups. A service evaluation of vitamin D testing and biochemical osteomalacia in the North-East of Scotland (57–58°N) could inform definitions and testing guidance.

**Methods:**

We identified adult 25OHD requests 8/7/2008–29/2/2020 and albumin-adjusted serum calcium (aCa), parathyroid hormone (PTH) and alkaline phosphatase (ALP) within 6 months of 25OHD testing. After excluding renal impairment and liver disease, we defined biochemical osteomalacia as ALP >130 IU/L and aCa <2.0 mmol/L and elevated PTH >9.2 or >6.8 pmol/L, depending on the assay. Possible biochemical osteomalacia was defined as 2 of these abnormalities in the absence of the third measurement. From these cases anonymised clinical data were then examined to confirm the diagnosis of osteomalacia.

**Results:**

25,379 eligible patients had 25OHD measured: 25% were <25 nmol/L (6,258/25,379) and 18% <20 nmol/L (4,536/25,379). 0.5% (126/25,379) of eligible patients had biochemical or possible biochemical osteomalacia. After reviewing clinical records, only 0.1% (29/25,379) had clinically confirmed osteomalacia, equivalent to 2–3 cases/y for a population of 0.5 million, none of the untreated cases of clinically confirmed osteomalacia had 25OHD >25 nmol/L. For the entire tested population, when 25OHD was <25 nmol/L untreated osteomalacia confirmed by clinical records was rare (0.4%).

**Conclusions:**

Osteomalacia is rare in North-East Scotland. Our data call into question designating 25OHD 25–50 nmol/L ‘insufficiency’. The risk of osteomalacia even when 25OHD is <25 nmol/L is very low.

## Introduction

2022 UK National Health and Care Excellence (NICE) guidance states that there is an increased risk of vitamin D deficiency if serum 25-hydroxyvitamin D (25OHD) is <25 nmol/L, with insufficiency defined as 25–50 nmol/L.^1^ The Scientific Advisory Committee on Nutrition’s (SACN) 2016 report, which informed NICE’s guidance, showed that around a fifth of UK adults have 25OHD <25 nmol/L, and stated that 25 nmol/L represented a ‘population protective level’, the concentration that individuals in the UK should be above, throughout the year, in terms of protecting musculoskeletal health.^2^ SACN noted that for osteomalacia ‘There was no clear serum 25(OH)D threshold concentration below which risk of osteomalacia increased but individual concentrations were <20 nmol/L in case reports and mean concentrations were ≤15 nmol/L in cross-sectional analyses’.^2^

The US Institute of Medicine ‘noted with some concern that serum 25OHD cut-points defined as indicative of deficiency for vitamin D have not undergone a systematic, evidence-based development process’, and that ‘This committee’s review of data suggests that persons are at a risk of deficiency relative to bone health at serum 25OHD levels of below 30 nmol/L. Some, but not all, persons are potentially at a risk for inadequacy at serum 25OHD levels between 30 and 50 nmol/L’.^3^

The US Endocrine Society had even higher cut-offs, defining deficiency as <50 nmol/L and sufficiency as ≥75 nmol/L, but in 2024 withdrew their endorsement of those definitions based on the failure of clinical trials to demonstrate benefits according to those cut-offs.^4^

We have previously shown an inexorable increase in vitamin D testing and prescribing in the UK, despite little change to very low numbers of annual cases of osteomalacia (30–100/y for 1998–2018 in England).^5^ However, hospital admission data, on which these numbers are based, may be an underestimate because they fail to capture cases managed in the community and missed diagnoses. As Scotland is further north than England, cases of osteomalacia might be higher.

NICE advises not to routinely test vitamin D in people who are asymptomatic.^1^ Testing is advised if there are musculoskeletal symptoms that may be attributable to vitamin D deficiency – suspected osteomalacia and chronic widespread musculoskeletal pain with other features of osteomalacia, and prior to treatment of bone disease with potent antiresorptive agents. People with osteoporosis treated with vitamin D and oral antiresorptive agents are not recommended for routine vitamin D testing.^1^

Osteomalacia is the only adult bone disease unequivocally linked to vitamin D deficiency. Vitamin D supplementation does not prevent fractures in community dwelling older people.^6^ Bone remodelling physiology and clinical experience suggest that 25OHD needs to be considerably below 25 nmol/L for prolonged periods for osteomalacia to occur. In our previous study in Auckland, New Zealand, only 0.23% of patients with 25OHD <25 nmol/L had osteomalacia, all below ≤18 nmol/L and around half of those below the limit of assay detection.^7^

Given the lack of strong evidence for the effect of vitamin D on anything other than osteomalacia and rickets,^6,8^ the known 25OHD assay variability,^9^ and disagreement about 25OHD interpretation, why are so many tests undertaken?

As the one NHS lab covering North-East Scotland, with ever increasing test requests, we gained permissions to undertake a service evaluation of all vitamin D requesting. We investigated the prevalence of biochemical osteomalacia (hypocalcaemia, secondary hyperparathyroidism, raised alkaline phosphatase), 25OHD status and risk of osteomalacia, and whether these might be used to provide guidance on test requesting and interpretation.

## Materials and methods

We analysed all 25OHD results from vitamin D samples collected between 8/7/2008 and 29/2/2020 using de-identified laboratory data from NHS Grampian (NHSG) (catchment population ∼0.5million, approximate latitude 57–58°N) in Scotland. All vitamin D samples from NHSG were sent to one laboratory: North Glasgow Biochemistry, Glasgow Royal Infirmary, NHS Greater Glasgow and Clyde. Samples were analysed by in-house tandem mass spectrometry from July 2008 to April 2012, and from April 2012 by immunoassay on Abbott Architect/Alinity. From 1/1/2011 25OHD requests were limited by Glasgow Royal Infirmary to one annually per patient to manage demand, unless discussed with NHSG clinical biochemistry.

We identified relevant biochemical test results ≤6 months before or after the index 25OHD test, and also those closest to the index test: adjusted serum calcium (aCa, reference interval 2.20–2.60 mmol/L), parathyroid hormone (PTH, reference interval 1.7–9.2 pmol/L until 1/6/2016, thereafter 1.3–6.8 pmol/L), alkaline phosphatase (ALP, reference interval 30–130 U/L), other liver function tests (GGT, ALT, AST), and serum creatinine. Adjusted serum calcium was calculated using: aCa = total calcium +0.02 × (40 – albumin g/L). We excluded any 25OHD results associated with renal impairment (serum creatinine ≥130 µmol/L), elevation of hepatic transaminases, or hypercalcaemia (aCa>2.60 mmol/L) during the index period. Elevated transaminases were defined as any of GGT >38 U/L for females, GGT >55 U/L for males, or AST >55 U/L or ALT >45 U/L.

We defined biochemical osteomalacia when all 3 of an elevated ALP (>130 IU/L), low aCa (<2.0 mmol/L), and elevated PTH (>9.2 or >6.8 pmol/L depending on assay) occurred within the index period. We defined possible biochemical osteomalacia as the presence of 2 of these 3 abnormalities during the index period and no measurement of the other test.

Only the first occurrence of a 25OHD measurement for a patient was considered in analyses.

Temporal graphs of ALP, aCa, and PTH for all the cases of biochemical osteomalacia or possible biochemical osteomalacia were constructed. From all cases of biochemical osteomalacia or possible biochemical osteomalacia, irrespective of 25OHD, we then examined clinical and laboratory data to decide whether there was clinical confirmation of osteomalacia ‘clinically confirmed osteomalacia’. Limited, anonymised data were extracted from laboratory data, clinical correspondence and hospital notes, and prescribing where available: sex, age, ethnicity, serum CRP and magnesium within 1 week of index 25OHD, source of request (community or hospital), residence (institution or own home), whether nutritional state was a concern (yes or no/unknown), whether chronic gastrointestinal or pancreatic disease was present (yes or no/unknown), and relevant diagnostic details, date of death. Prescription data were usually not available from hospital records unless provided in correspondence, for example, GPs’ referral letters, hospital discharge information, and clinical correspondence. Limitations in available records meant that it was often not possible to establish whether patients were being treated with vitamin D. Graphs and anonymised data were independently examined by two investigators (with reference to a third in case of disagreement) to produce a final categorisation of clinically confirmed treated osteomalacia (i.e. taking vitamin D at the time of 25OHD assessment), clinically confirmed osteomalacia (patient could not be found to be taking vitamin D at the time of 25OHD assessment), possible clinical osteomalacia or not clinical osteomalacia.

Decisions on final categorisation were based on one or several of the following: clinical staff documenting osteomalacia; timescale and pattern of biochemistry, including response to any vitamin D supplementation; alternative explanations for acutely deranged biochemistry, for example, bone metastases treated with potent bisphosphonates; severe sepsis or critical illness often with multimorbidity; malignancy, including treatment with chemotherapy; and liver disease.

The project was defined as a service evaluation and gained NHSG Caldicott Guardian approvals for both initial data acquisition and extraction of limited anonymised clinical data. Research ethics committee approval was not required because this was a service evaluation project.

Descriptive statistics (median and interquartile range (IQR) or proportions) are presented, as appropriate. Histograms of 25OHD values were plotted and 25OHD values by sex, age, time period, and classification of osteomalacia status were tabulated. The relationships between 25OHD decile and aCa, PTH, and ALP were examined, and boxplots were constructed. All analyses were conducted with Stata 16.

## Results

**Figure 1.**
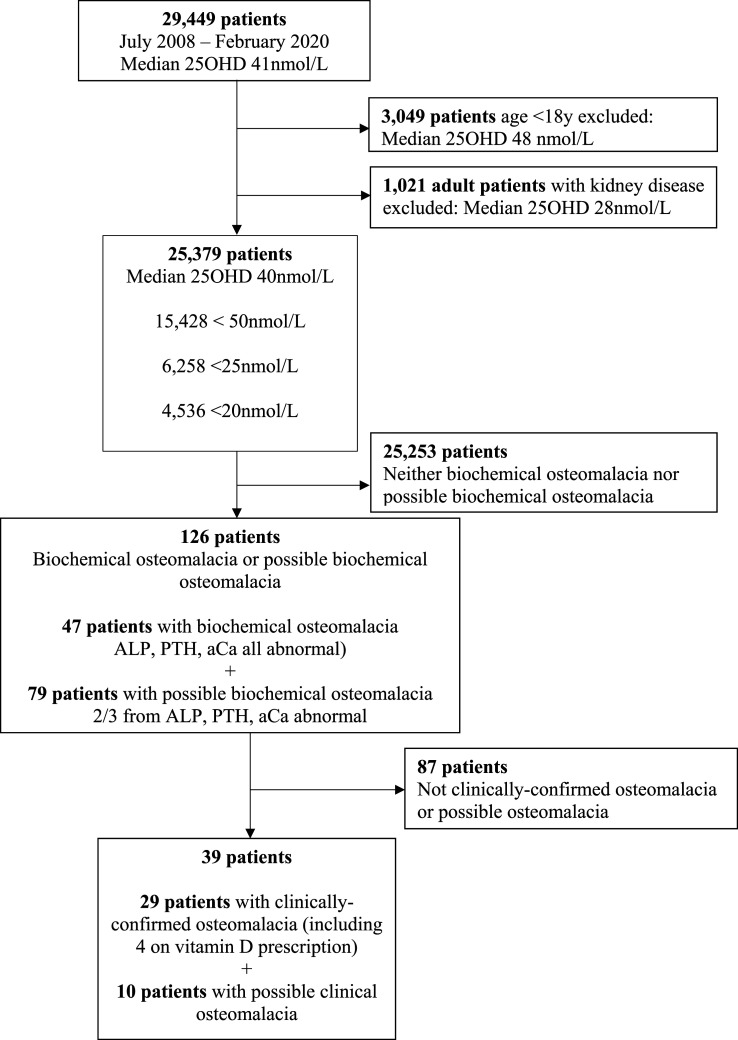
Flow chart for patients’ test data handling.

**Figure 2.**
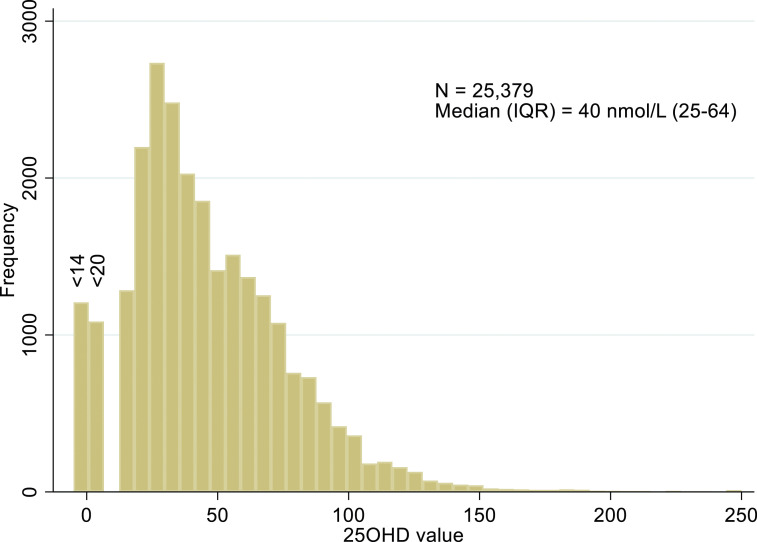
Histogram of 25OHD (nmol/L) in patients meeting inclusion criteria. Note 0 indicates a result less than a variable lower limit of quantification, either <14 or <20 nmol/L.

25OHD measurements were identified from 29,449 individuals, 26% male and 74% female, median 41 nmol/L (inter-quartile range, IQR, 25–64). Figure 1 provides the flow chart for patients’ test data handling. Removal of data from patients aged under 18 (N = 3,049, median age 9, 47% male, median 25OHD 48 [IQR 31–67]) and adults with kidney disease (N = 1,021, median age 74, 51% female, median 25OHD 28 [IQR 15–50]), left 25,379 individuals (86% of 29,449).The median value of 25OHD for these individuals included in the analysis was 40 (IQR 25–64) nmol/L (Figure 2), little different from the entire cohort.

Table 1 presents the sex, age, and time frame distribution for eligible 25OHD measurements. Median 25OHD differed little by sex, age, or date of sampling tertile, although there was a slight increase of median 25OHD with age up until 79 years. Between 2008–2012 and 2016–2020, there was a 400% increase in the number of participants tested for 25OHD status.

**Table 1.** 25OHD by sex, age, and timeframe for the included population.

		25OHD nmol/L		
	Frequency	Median	25^th^ percentile	75^th^ percentile
Sex^a^				
Female	18,756	41	25	66
Male	6,620	38	24	59
Age y^b^				
18–29	3,303	38	24	59
30–39	3,798	38	24	60
40–49	4,254	39	25	62
50–59	4,714	42	26	64
60–69	3,992	43	26	67
70–79	3,285	44	26	70
80+	2,031	37	21	67
Date range				
8/7/2008–25/5/2012	3,084	41	23	65
26/5/2012–11/4/2016	6,677	38	25	59
12/4/2016–29/2/2020	15,618	42	25	66

^a^3 missing sex.

^b^2 missing age.

For eligible patients, 61% (15,428/25,379) had 25OHD <50 nmol/L, 25% (6,258/25,379) <25 nmolL, and 18% (4,536/25,379) <20 nmol/L. 4.6% of patients (1171/25,379) had measurement of all three of aCa, PTH, and ALP in the time window.

Of 25,379 otherwise eligible results, 6,339 (25%) had none (5%) or only 1 of aCa, PTH or ALP (20%; of which, 94% had ALP measurement, 6% aCa, and 0% PTH). Even when 25OHD was <25 nmol/L, these measurements were uncommon: 6,259/25,379 (25%) of 25OHD were <25 nmol/L, but only 423 (7%) of these had all 3 measurements, 4,508 (72%) had 2 of 3 measurements, 1083 (17%) had 1 of 3 measurements (95% ALP, 5% Ca, 0% PTH), and 245 (4%) had none of the measurements during the index period.

**Figure 3.**
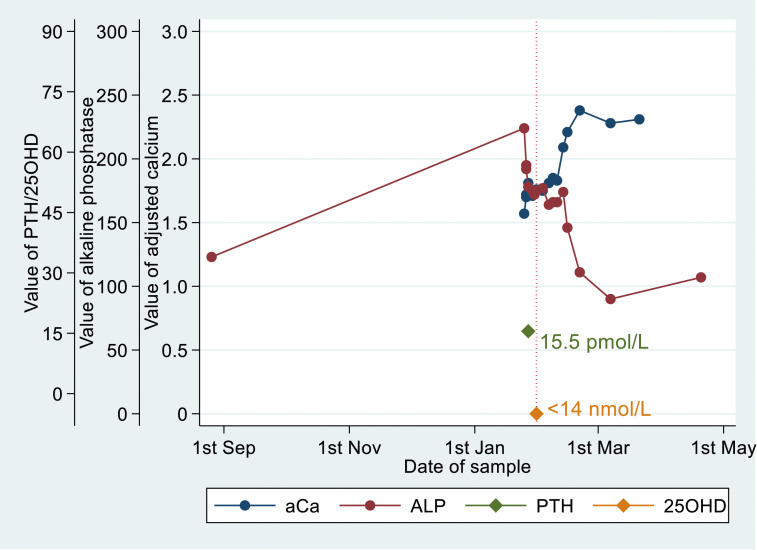
Laboratory values over time in a single illustrative patient with biochemical osteomalacia, with 25OHD <14 nmol/L, with preceding raised PTH of 15.5 pmol/L, low adjusted calcium and raised ALP. ALP and adjusted calcium return to reference intervals, after low 25OHD has been treated.

Table 2 provides details of the numbers of patients identified with biochemical osteomalacia and possible biochemical osteomalacia, using ALP, PTH, or aCa values within 6 months of the index 25OHD. Table 2 also provides the final clinical classification after temporal graphs of biochemical data were reviewed with anonymised clinical record data. An example of a graph illustrating biochemical osteomalacia is given in Figure 3:

**Table 2.** 25OHD values by biochemical osteomalacia classification.

25OHD nmol/L	^a^Biochemical osteomalacia within 6 month time window	^b^Possible biochemical osteomalacia within 6 month time window	Total
<20	28	38	
20–29	8	12	
30–50	10	15	
>50	1	14	
Total	47	79	126
Final classification			
Clinically confirmed osteomalacia	21 (45%)	8 (10%)	29
Possible clinical osteomalacia	2 (4%)	8 (10%)	10
Not clinical osteomalacia	24 (51%)	63 (80%)	87

^a^PTH, aCa, and ALP all abnormal irrespective of 25OHD.

^b^Two of PTH, aCa, and ALP abnormal irrespective of 25OHD.

126/25,379 (0.5%) eligible individuals tested were identified from biochemical testing as having biochemical or possible biochemical osteomalacia. After review of their clinical details, 25 were categorised as clinically confirmed osteomalacia, 4 as treated clinically confirmed osteomalacia, 10 as possible clinical osteomalacia, and 87 had an alternative diagnosis (not clinical osteomalacia). Thus, over nearly 12 years, only 29 of the eligible population of 25,379 (0.1%) of individuals who had 25OHD determined were found to have clinically confirmed osteomalacia. Even including cases of possible clinical osteomalacia only changes this figure to 0.2% (39/25,379). Analyses were repeated using the closest values which reduced the number of total biochemical cases identified by half to three-quarters but identified 23/29 of clinically confirmed cases and 10/10 cases of possible clinical osteomalacia.

Table 3 provides additional details on these 29 cases compared to 87 cases with alternative diagnoses. Patients with clinically confirmed osteomalacia were more likely to be female or aged ≥65, or were coded by the NHS as Black or Asian.

**Table 3.** Final classification and characteristics of the 126 patients with clinically confirmed osteomalacia or possible osteomalacia. Data presented as *n* (%).

	Clinically confirmed osteomalacia N = 29	Possible clinical osteomalacia N = 10	Not clinical osteomalacia N = 87
Male	9 (31)	4 (40)	36 (41)
Female	20 (69)	6 (60)	51 (59)
≥65y	19 (66)	8 (80)	45 (52)
<65y	10 (34)	2 (20)	42 (48)
White	18 (62)	8 (80)	75 (86)
Black or Asian	6 (21)	1 (10)	0 (0)
Unknown ethnicity	5 (17)	1 (10)	12 (14)
25OHD nmol/L			
<20	21 (72)	7 (70)	38 (44)
Of which <14 where measured	14 (48)	4 (40)	18 (21)
20–50	8^a^ (28)	3 (30)	34 (39)
>50	0 (0)	0 (0)	15 (17)
Source of 25OHD request			
Hospital	23 (79)	7 (70)	74 (85)
Community	6 (21)	3 (30)	13 (15)
Place of residence			
Institution	6 (21)	0 (0)	5 (6)
Own home	23 (79)	10 (100)	82 (94)
Died <6 months after 25OHD request			
Yes	7 (24)	4 (40)	22 (25)
No	22 (76)	6 (60)	65 (75)
Nutritional state a concern			
Yes	9 (31)	6 (60)	39 (45)
No/unknown	20 (69)	4 (40)	48 (55)
Chronic gastrointestinal or pancreatic disease			
Yes	12 (41)	6 (60)	36 (41)
No/unknown	17 (59)	4 (40)	51 (59)

^a^4 patients with treated osteomalacia, 25OHD: 37, 33, 41, 45 nmol/L; other patients 25OHD: 20, 20, 22, 24 nmol/L.

72% of patients with clinically confirmed osteomalacia not obviously on vitamin D treatment at the time of assessment had 25OHD <20 nmo/L; where measured, 48% had 25OHD <14 nmol/L. All patients with clinically confirmed osteomalacia not known to be on treatment had 25OHD <25 nmol/L and half of these were <14 nmol/L When 25OHD was <20 nmol/L, only 21/4,536 (0.5%) had untreated clinical osteomalacia. When 25OHD was <25 nmol/L, only 25/6258 (0.4%) had untreated clinical osteomalacia.

Most 25OHD requests for the 126 eligible individuals identified by biochemical testing came from hospital settings, and most patients had been living in their own homes. 24% of patients with osteomalacia died within 6 months of the 25OHD request, reflecting the fact that a high proportion of these patients were very frail older people living in their own homes, with multiple long-term conditions evident in clinical records. Clinical data indicated that 31% of cases had a concerning nutritional state, and 41% of cases had chronic gastrointestinal or pancreatic disease.

The relationships between deciles of 25OHD, and aCa, PTH, and ALP for the eligible index 25OHD measurements are shown in the supplemental Figure. Decreases in median aCa or increases in ALP, with decreasing 25OHD were small, although more evident when 25OHD was <20 nmol/L. Median PTH when 25OHD was <20 nmol/L was 11.4 (IQR 6.3–21.1), more than 50% higher than the median PTH values for 25OHD ≥20 nmol/L [7.3(3.6–13.3)]. There was a trend for the median PTH to be higher in the 25–50 range than >51 nmol/L. Data relate to two different PTH assays with reference ranges of 1.7–9.2 pmol/L and 1.3–6.8 pmol/L.

## Discussion

Between 2008 and early 2020 around 5% of all adults in the North-East of Scotland had 25OHD measured at least once, with numbers tested increasing four-fold over time, despite limiting requests to one per patient/year. Three-quarters of patients were women and test results showed little variation by age group. This might suggest that older people at a risk of osteomalacia are being missed or are already receiving supplementation, for example, guidance for care homes in Scotland.^10^ This might also suggest that patients who are otherwise well, but nevertheless have particular concerns about vitamin D status, are more likely to be tested. After a reduction in testing during the early stages of the COVID-19 pandemic, testing from 2023 has returned to the pre-pandemic trajectory.

The median 25OHD of 40 nmol/L of the population included in the analysis differed little from the overall population or for sex, age, or date of sampling. However, patients with renal impairment (here defined as serum creatinine ≥130 µmol/L) had a lower median 25OHD of 28 nmol/L.

In this population undergoing vitamin D testing 25% had 25OHD <25 nmol/L and 18% <20 nmol/L. Of all people tested, only 0.1% had osteomalacia, and even when 25OHD was <25 nmol/L or <20 nmol/L osteomalacia was rare (0.4% and 0.5% respectively). 48% of people with osteomalacia had 25OHD <14 nmol/L, the lower limit of assay detection for part of the investigation period. Only 7% of patients with 25OHD <25 nmol/L had all of aCa, ALP, and PTH measured in the 6-month time window. The lack of PTH testing may have been because osteomalacia was not under consideration, although lack of knowledge to test and limited availability for primary care may also have contributed. Median PTH was slightly higher when 25OHD was in the 25–50 nmol/L range compared to ≥51 nmol/L.

We identified around 2–3 cases/y of osteomalacia in the North-East of Scotland. Applying these results to Scotland as a whole equates to around 25 cases/y (although most of the Scottish population lives further south). Previously, we showed 30–100 cases/y for England for a population about 10 times the size of Scotland.^5^ Although Scottish figures are therefore higher, they still represent very small numbers of cases of osteomalacia, and a tiny fraction of people with 25OHD <25 nmol/L. We found no evidence that people were at a risk of osteomalacia with 25OHD 25–50 nmol/L.

In our previous study^7^ with similar methods conducted with laboratory data from Auckland, New Zealand, the median 25OHD was higher at 63 nmol/L. This is unsurprising given Auckland’s lower latitude (35^o^  S). Of those tested in Auckland, 8% were <25 nmol/L, compared to 25% in the North-East of Scotland. When 25OHD was <25 nmol/L, 0.2% had a diagnosis of osteomalacia in Auckland, compared to 0.4% in the North-East of Scotland. All cases of osteomalacia in Auckland had 25OHD ≤18 nmol/L, half of these undetectable. In the North-East of Scotland, all patients with osteomalacia not known to be on treatment had 25OHD <25 nmol/L with half of these <14 nmol/L.

Analytical performance of 25OHD assays continues to be challenging. A recent paper from the Vitamin D External Quality Assessment Scheme (DEQAS) highlights continuing performance issues, with none of the methods evaluated consistently meeting the performance criteria of ±5% bias and <10% coefficient of variation, including Abbott Alinity and Architect used here.^9^ Under-recovery of 25OHD_2_ from supplement use continues to be challenging. Although this is a less bioactive form, this increases the risk of classifying people at an increased risk of deficiency by NICE’s current criteria. Cross-reactivity with other vitamin D analytes is also an issue in immunoassays.

Our data rely on adults who had 25OHD testing, together with aCa, ALP, or PTH. We did not include low serum phosphate in our assessment, since it was unhelpful in our previous study, where serum phosphate, where measured, was in the reference range for 7/8 cases of osteomalacia.^7^ Another study showed that plasma phosphate was associated with false negative rates of 81–96% for osteomalacia.^11^ We excluded people with renal or liver disease from our investigation. An important limitation of this work is that many with 25OHD measurements did not have sufficient additional blood testing to identify biochemical osteomalacia. We did not include patients with no or only one biochemical test in our analysis – it seems unlikely that the requesting clinician would be considering osteomalacia for these patients, particularly when only 6% of single tests were serum calcium. Nevertheless, we have whole population data, so we have essentially identified all the cases in the population where testing was done to make identification possible. The true population incidence of osteomalacia is therefore likely to be higher than we have identified. However, data on prescriptions were often missing – so some patients with osteomalacia may already have been started on supplements.

Our data show that 25OHD testing in adults is prevalent and increasing. This appears unrelated to identifying osteomalacia based on test requests, which is rare. Since 2016 NHS advice, including in Scotland, has been for all adults to take 10 mcg/d of vitamin D from October to March and year round in high risk groups (pregnant and breastfeeding women, people with little or no sun exposure or from ethnic groups with dark skin).^12^ 25OHD testing should therefore be rarely needed if this advice is followed.

Our data also call into question designating 25OHD of 25–50 nmol/L as ‘insufficiency’, which implies pathophysiology, given that only 0.4% of people <25 nmol/L were found to have clinically confirmed osteomalacia, and none >25 nmol/L. Suppression of PTH was used to develop guidance on 25OHD optimisation, but as the 2024 Endocrine Society report states ‘the thresholds at which physiological changes occur (e.g. increases in PTH secretion) may or may not conform to the thresholds at which the risks for undesirable clinical outcomes become unacceptable’.^4^

Contrary to commonly held belief, vitamin D supplementation trials have not prevented osteoporotic fractures except in vitamin D deficient nursing home residents who were also supplemented with calcium.^6^ Newer trials in populations with good 25OHD status have not changed that finding.^4^ The most recent Cochrane review of population-based interventions was unable to demonstrate that vitamin D prevented falls in older people living in the community.^13^ There is moderate quality evidence that vitamin D supplementation for people with poor 25OHD status probably reduces the rate of falls but probably makes little or no difference to who falls in care homes or hospitals^14^ – people who are known to be at risk for deficiency through lack of sunlight, where supplementation is already advised in the UK.

The largest ever UK trial of calcium and/or vitamin D supplementation in older people after fracture, where mean baseline 25OHD was 37 nmo/L for the sample of patients assessed, found that vitamin D did not prevent fractures, infections, falls or reduce cancers, or deaths from cancer, heart disease or overall.^15–17^ There are, however, no adequately powered large scale randomised trials for these outcomes for vitamin D supplementation alone for people with 25OHD <25 nmol/L. Given that clinically confirmed osteomalacia when 25OHD <25 nmol/L appears rare, a case could be made for such a trial.

These data call into question guidance on 25OHD interpretation and the necessity of the vast majority of 25OHD test requests from both primary and secondary care. Existing NHS guidance properly implemented removes the necessity to test 25OHD in almost all cases, except suspected osteomalacia, chronic widespread pain with other features of osteomalacia such as proximal muscle weakness, and known bone disease prior to potent antiresorptives.^1^ Food fortification could ensure that truly high risk groups, such as frail older people living in their own homes, have adequate vitamin D status.^18,19^ For example, the introduction of fortification of fluid milk products in Finland reduced the prevalence of 25OHD <30 nmol/L from 13.7% to 0.9% for the population who did not take vitamin D supplements.^20^ Widespread vitamin D testing and supplementation does not seem a good use of scarce NHS resources.

## Supplemental Material

Supplemental Material - Biochemical osteomalacia in adults undergoing vitamin D testing in the North-East of Scotland Supplemental Material for Biochemical osteomalacia in adults undergoing vitamin D testing in the North-East of Scotland by Angus D Macleod, Mark J Bolland, Andrew Balfour, Andrew Grey, Josh Newmark, and Alison Avenell in Annals of Clinical Biochemistry
