# Supplementary material for: Biochemical osteomalacia in adults undergoing vitamin D testing in the North-East of Scotland
Source: Ann Clin Biochem. 2025 Jan 25;62(4):303–11. doi: 10.1177/00045632251315671 (PMC12227805; doi:10.1177/00045632251315671)
Supplement: Supplemental Material - Biochemical osteomalacia in adults undergoing vitamin D testing in the North-East of Scotland [file sj-pdf-1-acb-10.1177_00045632251315671.pdf]

Supplementary Figure

Box and whisker plots with distribution of adjusted calcium (mmol/L), alkaline phosphatase (IU/L), parathyroid hormone (pmol/L) with 25OHD (nmol/L) for eligible cohort. Centre line in each box represents 50th percentile (median) value. Bottom and top of each box represent 25th and 75th percentile values. Upper and lower “whisker” represent upper adjacent value and lower adjacent value. Outlying values not shown. Numbers of patients in each 25OHD category: <20nmol/L 3,922; 20-24nmol/L 2,337; 25-29nmol/L 2,252; 30-35nmol/L 2,482, 36-42nmol/L 2,359; 43-50nmol/L 2,369; 51-59nmol/L 2,340; 60-70nmol/L 2,361; 71-85nmol/L 2,339; >85nmol/L 2,618.

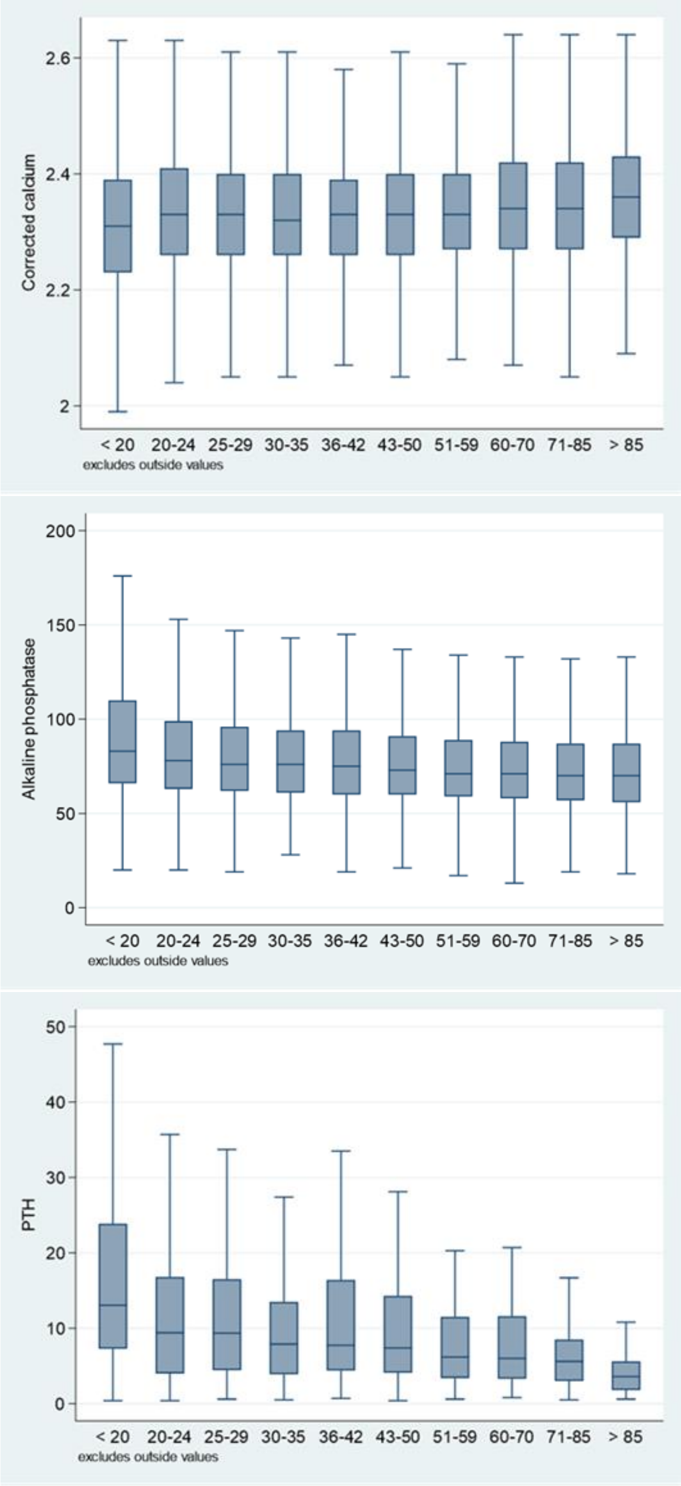

Supplementary table

Closest PTH, alkaline phosphatase and adjusted calcium values to 25OH vitamin D for 29 clinical confirmed patients with osteomalacia. Patients usually had multiple values for adjusted calcium and alkaline phosphatase; in some cases closest values did not exceed our cut-offs.

Adjusted serum calcium (aCa, reference interval 2.20-2.60 mmol/L), parathyroid hormone (PTH, reference interval 1.7-9.2 pmol/L until 1/6/2016, thereafter 1.3-6.8 pmol/L), alkaline phosphatase (ALP, reference interval 30-130 U/L). T = on vitamin D treatment. N/A = not available.

Patient	Date of 25OH vitamin D	Index 25OH vitamin D nmol/L	Closest alkaline phosphatase	Closest PTH	Closest adjusted calcium
1	19-Sep-15	37 T	145	49.3	1.93
2	27-Jan-12	<14	175	15.5	1.76
3	18-Jan-15	22	234	31.7	1.90
4	09-Nov-09	14	146	68.6	1.89
5	11-May-17	<14	716	66.3	1.66
6	09-Jan-15	<20	241	92.2	1.95
7	13-Mar-09	<14	169	50.5	1.91
8	06-May-14	33 T	342	43.2	2.32
9	08-Feb-10	14	243	50.5	1.97
10	22-Feb-12	<14	369	42.9	1.45
11	19-Dec-18	<14	831	88.9	1.34
12	13-Jun-11	<14	1284	152	1.96
13	19-Sep-15	<14	157	44.8	2.00
14	29-Oct-15	20	135	21.8	1.63
15	20-Jun-17	<14	156	25.3	1.54
16	26-Mar-15	41 T	254	114	1.64
17	01-Feb-18	<14	295	N/A	1.91
18	26-Jan-15	<20	325	N/A	1.98
19	29-Jan-14	<20	1016	N/A	1.42
20	06-Mar-14	<20	219	N/A	1.77
21	28-May-14	20	177	N/A	1.86
22	20-Apr-15	<20	339	N/A	1.99
23	28-Jan-11	<14	448	N/A	1.80
24	08-Aug-09	<14	91	17.6	1.43
25	10-Feb-11	<14	1929	46.5	1.54
26	19-Sep-12	24	152	36.7	2.02
27	24-Nov-11	<14	120	39.9	1.13
28	04-May-19	<14	351	56.8	2.19
29	03-Sep-10	45 T	215	N/A	2.05
